# Persistent androgen receptor addiction in castration-resistant prostate cancer

**DOI:** 10.1186/s13045-015-0225-2

**Published:** 2015-11-13

**Authors:** Michael T. Schweizer, Evan Y. Yu

**Affiliations:** Division of Oncology, Department of Medicine, University of Washington/Fred Hutchinson Cancer Research Center, Seattle, WA 98109 USA

**Keywords:** Prostate cancer, Castration-resistant prostate cancer, Androgen receptor, Androgen receptor splice variant, Drug resistance, Signaling pathway

## Abstract

It is now understood that persistent activation of the androgen receptor (AR) signaling pathway often underlies the development of castration-resistant prostate cancer (CRPC). This realization led to renewed interest in targeting the AR and ultimately to the development of the potent next-generation AR-directed agents abiraterone and enzalutamide. While these drugs prolong survival in men with CRPC, they are unfortunately not curative. Perhaps not surprisingly, evidence points to persistent AR signaling as one of the key drivers by which resistances to these agents develops. In this context, activation of the AR signaling program can occur through a number of molecular adaptations, including alterations leading to persistent canonical AR signaling (e.g., AR amplification/overexpression, elucidations/concentration of intratumoral androgens), activation of the AR program via feedback pathways (e.g., AKT/mTOR/Pi3K, HER2/Neu), and activation of the AR program via mutation or substitution (e.g., AR ligand binding domain mutation; AR splice variants; glucocorticoid receptor signaling). This review will provide an overview of the more clinical relevant (i.e., druggable) pathways that have been implicated in the emergence of drug resistance in men with CRPC and highlight some of the ongoing efforts towards developing therapeutics to impair these mechanisms.

## Background

Androgen deprivation therapy (ADT) has remained the backbone of treatment for advanced prostate cancer since the remarkable palliative effects of surgical castration were first described by Huggins and Hodges back in the 1940s [1]. While ADT is effective at controlling metastatic prostate cancer, for the vast majority of patients, these benefits are short lived, and most will progress to the lethal phenotype of the disease, so called castration-resistant prostate cancer (CRPC).

CRPC is defined as progressive prostate cancer in the face of castrate serum testosterone levels (≤50 ng/dL) [2, 3]. Older literature has described this disease state as “hormone refractory”; however, the term “castration-resistant” has been adopted largely due to the increasing recognition that androgen receptor (AR) signaling still plays a vital role in driving prostate cancer growth and remains a viable target in this disease space [4]. Indeed, the development of newer drugs that function to inhibit ligand-AR interaction (e.g., abiraterone (an inhibitor of extragonadal androgen synthesis) and enzalutamide (a potent irreversible AR antagonist)) have provided proof of principle that the AR remains an important driver of CRPC growth [5–8].

Perhaps not surprising, more complete AR signaling inhibition with drugs like abiraterone and enzalutamide has not proven curative, with resistance typically emerging within a year of drug initiation [5–8]. Our understanding of resistance mechanisms operating to drive continued CRPC growth after receipt of either abiraterone or enzalutamide has clarified that persistent AR signaling is still one of the major drivers.

Persistent activation of the AR signaling program occurs both not only through molecular adaptations of the *AR* itself but also through a number of accessory oncogenic pathways promoting persistent AR activation, ultimately leading to progressive prostate cancer. Broadly, these mechanisms include alterations leading to persistent canonical AR signaling (e.g., AR amplification/overexpression, elucidations/concentration of intratumoral androgens), activation of the AR program via feedback pathways (e.g., AKT/mTOR/Pi3K, HER2/Neu), and activation of the AR program via mutation or substitution (e.g., AR ligand binding domain mutation, AR splice variants, glucocorticoid receptor signaling) [9–25].

Detailed reviews have been written on any one of these pathways, and our goal is not to catalog the numerous molecular adaptations that can precede the emergence of CRPC drug resistance. Rather, we seek to provide an overview of the more clinical relevant (i.e., druggable) pathways that have been implicated in the emergence of drug resistance and to highlight some of the ongoing efforts towards developing therapeutics to impair these mechanisms. This review will therefore focus on the evidence for several key mechanisms implicated in promoting sustained AR signaling, with an emphasis on those that may be targetable in the near term.

## Review

### Androgen receptor function and structure

**Fig. 1:**
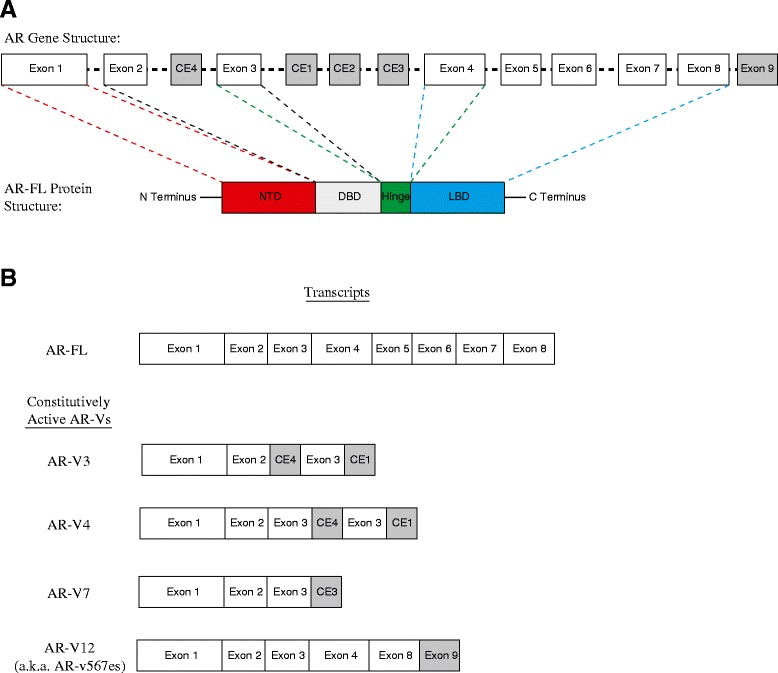
Androgen Receptor Structure. **a**. The *AR* gene is located on the X chromosome at position Xq11-Xq12. It is composed of eight exons, which encode for four regions: N-terminal domain (*NTD*), DNA binding domain (*DBD*), the hinge region and the ligand binding domain (*LBD*). **b**. Several cryptic exons (*CE*) as well as exon 9 are involved in the formation of several AR splice variants (AR-Vs) which lack the AR ligand binding domain. Four of these AR-Vs have been shown to possess constitutive activity (i.e., AR-V3, AR-V4, AR-V7, and AR-V12)

The AR is a nuclear transcription factor encoded on the X chromosome at position Xq11-Xq12 [26, 27]. It contains eight exons and is composed of four domains: the N-terminal domain (i.e., transcriptional activation domain) (exon 1), DNA-binding domain (exons 2 and 3), a hinge region (exons 3 and 4), and the ligand-binding domain (i.e., C-terminal) (exons 4–8) (Fig. 1). An overly simplistic model of canonical AR signaling involves: (1) androgen binding the AR ligand binding domain, (2) dissociation of chaperone proteins (i.e., heat shock proteins), (3) AR nuclear transport and dimerization (likely through microtubule interaction with the hinge region), (4) binding of dimerized AR to androgen response elements (ARE) located within the promoters of AR target genes, (5) recruitment of AR co-activators, and (6) transcription of AR target genes. A number of additional events, such as AR phosphorylation and interaction with other co-regulators and transcription factors likely also play a role in modulating transcription of AR target genes [28].

**Fig. 2:**
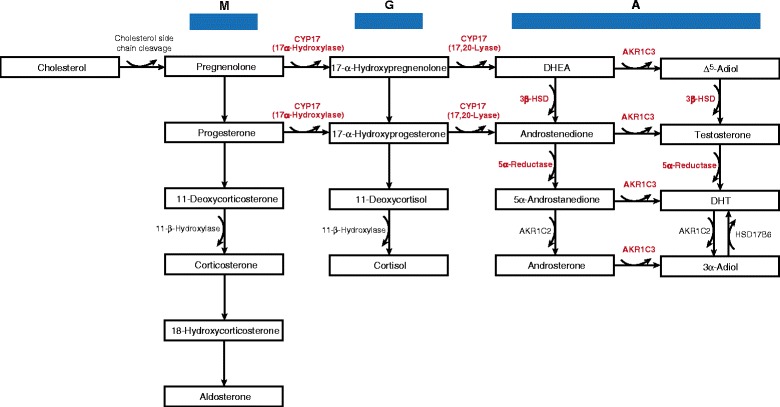
Steroidogenesis pathway. Enzymes shown to be upregulated in a castration-resistant state are highlighted *red. M* mineralocorticoid biosynthesis pathway, *G* glucocorticoid biosynthesis pathway, *A* androgen biosynthesis pathway

**Fig. 3:**
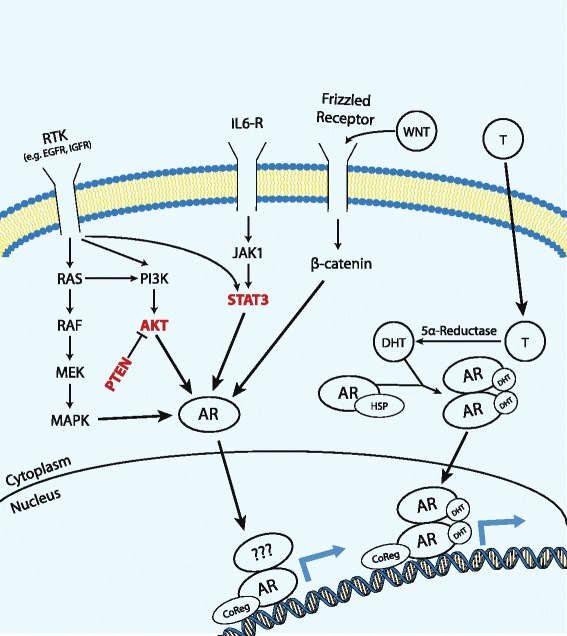
Androgen receptor activation pathways. AR transcription can occur either through canonical AR-FL signaling or through cross-talk between alternative signaling pathways. Ligand-independent AR transcription likely occurs through a number of mechanisms and may involve the formation of AR homodimers (as is the case with the AR-FL canonical signaling pathway) or heterodimerization between AR-FL, AR-Vs, and AR-mutants. Cross-talk between signaling pathways likely augment AR transcriptional activity in several ways, including through N-terminal phosphorylation, or by promoting AR nuclear translocation. Key nodes affected by several pathways are highlighted in *red. RTK* receptor tyrosine kinases, *T* testosterone, *HSP* heat-shock proteins, *IL6-R* IL-6 receptor, *CoReg* co-regulators

The *AR* represents perhaps the first described lineage-specific oncogene, with prostate cancer demonstrating a persistent addiction to AR- ignaling even in its late stages—a reflection of its emergence from normal prostatic epithelium [29, 30]. The survival of a given prostate cancer cell is tightly linked to persistent AR signaling, and as such, these malignant cells will undergo a number of adaptive changes to ensure persistent AR signaling. Reflective of the reliance of prostate cancer on the expression of AR target genes is the observation that over 70 % of CRPC cases harbor *AR* pathway aberrations, with AR transcriptional activity persisting in the majority of cases of CRPC [31].

### Persistent canonical AR pathway activation

The observation that AR-regulated genes (e.g., *PSA*) remain expressed in a castration-resistant state renewed interest in targeting the AR in men that had progressed on LHRH analogue therapy. Ultimately, this work led to the development of drugs that more effectively inhibit the ligand-AR interaction. Abiraterone, a CYP-17 inhibitor capable of inhibiting extragonadal testosterone synthesis (i.e., adrenal and intratumoral) was approved on the basis that it significantly prolongs survival in both the pre- and postdocetaxel spaces [6, 7, 32]. Approval for enzalutamide, a potent AR antagonist, was granted on the basis of a survival advantage in similar patient populations [5, 8]. The clinical efficacy of abiraterone and enzalutamide has provided proof of principal that canonical AR signaling is an important driver of CRPC growth that can be targeted for clinical gains.

Interestingly, a correlation between increased full-length AR (AR-FL) expression and *AR* copy number gains with the emergence of resistance to second generation AR-directed agents has been documented [9, 11, 33, 34]. This implies that persistent canonical AR signaling is likely engaged even in the presence of drugs that should otherwise be able to inhibit AR-FL from interacting with its ligand (i.e., androgens). This could be a result of pharmacokinetic issues whereby drugs are unable to reach sufficient concentrations within the tumor microenvironment or that intratumoral steroidogenesis is able to overcome the inhibitory effects of these agents [35, 36]. A more definitive explanation for this effect is needed and continues to be an area of active research.

#### AR overexpression and copy number alterations

One of the more commonly observed events as prostate cancer progresses from a hormone-sensitive to castration-resistant state is the adaptive upregulation of the AR, a finding supported by preclinical as well as clinical studies [13, 20]. Chen and colleagues demonstrated that a number of prostate cancer cell lines will adaptively increase their AR expression as they are passaged in castrate mice over time, and that this overexpression of AR is sufficient to induce resistance to the effects of surgical castration as well as treatment with the first-generation anti-androgen bicalutamide [13]. AR overexpression as a driver of resistance is also supported by data from rapid autopsy programs showing that AR expression is increased in patients that have died from CRPC [37, 38]. These findings provided a strong rationale for developing drugs like abiraterone and enzalutamide to target persistent AR signaling in men with CRPC.

In addition, an adaptive increase in AR expression and *AR* copy number gain has also been observed in circulating tumor cells (CTCs) as well as cell-free circulating tumor DNA (ctDNA) from patients with CRPC receiving next-generation AR-directed therapies (i.e., abiraterone and enzalutamide)—implicating this as mechanism by which cell growth can escape the inhibitory effects of these drugs [9, 11, 33, 34]. Perhaps more indicative of the importance AR overexpression plays in promoting CRPC growth comes from the results of a recently published, large metastatic biopsy program enrolling men with CRPC [31]. In that paper, 150 men with metastatic CRPC underwent biopsy and had sufficient tumor material to undergo integrative genomics analysis (i.e., whole exome and transcriptome sequencing). That study revealed that the most frequently observed genetic alteration was *AR* copy number gain, occurring in 45 % of cases.

#### Maintenance of intratumoral androgens

In addition to overexpressing AR, there is evidence that the maintenance of androgens within the tumor microenvironment may also be an important means by which canonical AR signaling drives castrate-resistant growth. In men with hormone-sensitive, localized prostate cancer, it has been found that intraprostatic androgens will fall by ~75 % following initiation of an LHRH analogue therapy, with residual androgen levels sufficient to drive expression of AR-activated genes such as PSA [39, 40]. In addition, Montgomery and colleagues have shown that compared to benign prostatic tissue and primary tumors from eugonadal patients, metastases in men with CRPC had significantly elevated testosterone levels [19]. Interestingly, increased mRNA expression of several steroidogenic enzymes (i.e., 3βHSD, CYP17A1, AKR1C3, and SRD5A2) accompanied the increased testosterone concentrations found within metastatic CRPC foci—providing a mechanistic explanation for why increased androgens levels are found within the metastatic tumor microenvironment (Fig. 2).

In support of the putative role steroidogenic enzymes play in sustaining intratumoral androgens and subsequent AR activation, cell culture and xenografts models of CRPC have demonstrated that AKR1C3-mediated steroidogenesis can lead to the emergence of resistance to next-generation AR-directed therapies [41, 42]. Further evidence for the importance that intratumoral androgen biosynthesis plays in promoting CRPC resistance is provided through experiments exploring the effects of impairing the activity of key steroidogenic enzymes. For instance, inhibiting AKR1C3, an enzyme involved in converting DHEA-S into the potent AR ligands testosterone and dihydrotestosterone (DHT), has been shown to inhibit prostate cancer cell growth in cell culture and xenograft models [43, 44]. In addition, a synergistic anti-tumor effect when indomethacin (an AKR1C3 inhibitor) is combined with either abiraterone or enzalutamide has been reported in otherwise resistant prostate cancer cell lines [41, 42]. Clinical trials to test the effects of inhibiting AKR1C3 are currently being developed.

The steroidogenic enzyme 3βHSD (encoded by either *HSD3B1* or *HSD3B2*) is able to catalyze the conversion of androstenediol to testosterone and also catalyzes the initial rate-limiting step in converting DHEA to DHT [45]. As noted above, 3βHSD mRNA levels are higher in metastases from men with CRPC and correlate with increased intratumoral androgen levels [19]. A gain-of-function mutation in 3βHSD has also been described that results in increased DHT production and may be selected for following chronic castration (i.e., in a castration-resistant state) [12]. This mutation may also represent a mechanism by which abiraterone resistance occurs, as increased 3βHSD activity can result in persistent adrenal steroidogenesis in spite of CYP17 inhibition. These observations have implicated 3βHSD as a means by which ligand is able to persist in spite of ADT, raising the specter that 3βHSD activity may be one of the mechanisms leading to castration resistance and indicating that 3βHSD may be a viable drug target [16].

Of note, abiraterone does function as a weak 3βHSD inhibitor, and it has been proposed that increased abiraterone exposure may result in more complete 3βHSD inhibition [8]. Dose-escalated abiraterone may therefore have the resultant effect of decreasing the metabolic flux to DHT and potentially reverse resistance to standard dose abiraterone. A clinical trial testing if dose-escalated abiraterone can further suppress intratumoral androgen levels (presumably through impairing 3βHSD activity) is currently underway (clinicaltrials.gov: NCT01503229). Interestingly, 3βHSD has also been shown to catalyze the conversion of abiraterone to Δ^4^-abiraterone (D4A), a more potent compound able to inhibit several steroidogenic enzymes and antagonize the AR to a degree comparable to enzalutamide [17]. Whether a resistant phenotype emerges following treatment with abiraterone may be in part due to the degree to which 3βHSD is able to promote DHT vs*.* D4A production. More work to understand the relative contribution that DHT and D4A synthesis plays in promoting abiraterone response/resistance is needed.

Enhanced androgen and hormone substrate uptake into the tumor microenvironment may be another explanation accounting for the higher concentration of androgens found within metastatic foci. Androgen uptake is mediated by the OATP (organic anion transporting polypeptide) transporter and polymorphisms in the *SLCO* family of genes encoding the OATP transporters have been found to associate with outcomes [22, 23, 46–49]. For instance, SNP alleles associated with enhanced androgen transport or increased *SLCO* expression have been correlated with increased prostate cancer-specific mortality and higher rates of disease progression [22, 23, 46, 48]. Preclinical studies have shown that statins are able to competitively bind SLCO2B1 and prevent uptake of the testosterone precursor DHEAS [23, 50]. Retrospective data has also shown a correlation between statin use and prolonged time to progression [50]. Whether this correlation is due to preventing androgen uptake by the OATP transporter SLCO2B1 or due to decreasing cholesterol—an essential substrate for all steroid hormones—remains to be seen [51].

### AR mutations, variants, and alternate hormone activation

In addition to activating the AR transcriptional program via canonical full-length AR signaling, variants of the AR that occur through alternative splicing or mutations in the ligand binding (i.e., C terminal) domain of the AR are also able to keep AR signaling engaged in a castration-resistant state [9, 11, 20, 52–56]. In addition, other nuclear hormone receptors, such as the glucocorticoid receptor (GR), are able to activate a seemingly similar transcriptional program to the AR and may be a mechanism by which prostate cancer cells develop resistance to drugs that block AR signaling by disrupting the AR ligand interaction [57–60].

#### AR point mutations

A number of point mutations within the ligand-binding domain of the AR have been described that result in resistance to first-generation anti-androgens (e.g., flutamide, nilutamide, and bicalutamide) [61, 62]. Some of these mutations result in increased AR activity upon exposure to these agents, likely explaining why some men experience an anti-androgen withdraw effect following their cessation [63]. Similarly, the AR mutation F877L has been found to associate with resistance to the second-generation AR antagonists enzalutamide and ARN-509, resulting in AR activation upon exposure to these agents [34, 64–66]. More recently, preclinical models have demonstrated that the AR antagonist ODM-201 is able to inhibit F877L prostate cancer mutant cell growth [67]. Recent phase 1/2 testing has demonstrated that ODM-201 is well tolerated and is active in men with CRPC [68]. This study did not, however, assess for the presence of the F877L mutation, and ODM-201’s activity in this subset of patients remains undefined.

#### AR splice variant

Over the last few years, it has become increasingly well recognized that in addition to point mutations, alternative splicing events that lead to constitutively active AR variants (AR-Vs) are another clinically relevant means by which prostate cancer is able to progress in spite of agents that effectively disrupt the AR-ligand interaction (Fig. 1) [9, 20, 25, 31, 53–55, 69]. The AR-Vs, the most commonly occurring AR-V being AR-V7, retain the ability to activate their transcriptional program in spite of lacking the AR ligand-binding domain [70]. A recent prospective biomarker trial reported by Antonarakis et al*.* found an association between the emergence of AR-V7 mRNA transcripts, as measured from CTC, and resistance to abiraterone and enzalutamide [9]. This data provides an elegant biologic rationale for why drugs that interfere with the AR-ligand interaction may not be effective in patients harboring certain AR-Vs at significant levels; however, it should be noted that increased AR-FL expression always accompanied the emergence of AR-V7 transcripts and was also found to associate with drug resistance (albeit not as strongly as the presence of AR-V7) [20, 52–56]. Interestingly, AR-V7 does not appear to be predictive for lack of response to docetaxel—providing a rationale for precision oncology trials stratifying patients between AR-directed therapies and chemotherapies on the basis of AR-V status [71].

Recently, Steinestel et al. reported their experience using a method similar to the one employed by Antonarakis and colleagues for detecting AR-V7 transcripts [72]. They also found that the presence of AR-V7 transcripts associated with a lack of PSA response to abiraterone or enzalutamide; however, it should be noted that one out of five AR-V7-positive patients in this trial had a PSA response (i.e., ≥50 % decline in PSA from baseline) to abiraterone, which is in conflict with the results reported by Antonarakis et al. Needless to say, further studies to validate the utility of detectable AR-V7 transcripts as a predictive and/or prognostic biomarker, ideally in the context of a randomized therapeutic trial, are needed. In addition, mechanistic studies are need in order to determine if AR-Vs are drivers of resistance or merely indicative of a larger resistance program being at play.

The N-terminal domain of the AR is critical for AR transactivation and subsequent activations of its transcriptional program [73]. As noted above, deletion of the C-terminal (i.e., ligand-binding domain) may result in constitutive AR activity, and as such, there are a number of ongoing efforts to develop drugs capable of inhibiting the N-terminal domain or its co-activators/epigenetic regulators, as a means to overcome AR-V-mediated drug resistance [10, 18, 74]. In addition to directly targeting the N-terminal domain, drugs intended to suppress AR-V expression are also being developed [75–78].

Due to the fact that the AR N-terminal is inherently unstructured, developing drugs that block AR-signaling through impairing N-terminal domain activity is not trivial [79]. Epi-001 and its analogues (e.g., Epi-506) are small molecule antagonists designed to target the AR N-terminal domain. They reportedly covalently bind to the N-terminal domain, thus inhibiting the protein-protein interactions required for AR-mediated transcription [80]. Importantly, these N-terminal domain antagonists have been shown to retain activity in prostate cancer cell lines expressing AR-Vs. Of note, Epi-001 may have a broader mechanism of action than first described [81]. It has been shown to have general thiol alkylating activity, resulting in multi-level effects on prostate cancer cells. In addition to covalently binding to the AR N-terminal domain, Epi-001 has been shown to inhibit transcription of the *AR* gene and to modulate PPARγ activity. Likely as a result of these diverse mechanisms of action, Epi-001 has also been shown to inhibit the growth of AR-null prostate cancer cell lines. A phase I/II trial testing Epi-506, a pro-drug of Epi-001, is planned to open in the near future [74].

The AR transcriptional machinery is immensely complex and involves not only AR binding to the enhancer elements of target genes but also the assembly of RNA polymerase II with a number of co-activator proteins [31, 82–84]. Much work has gone into understanding the key regulators of AR-mediated transcription, in an effort to identify novel therapeutic targets. Genetic knockdown of HDAC1 or HDAC3 or treatment with an HDAC inhibitor has been shown to prevent the assembly of the AR-transcriptional complex and prevent expression of AR-mediated genes [84]. Unfortunately, a small phase II study testing the HDAC inhibitor romidepsin failed to demonstrate sufficient activity in patients with CRPC [85]. Another strategy by which AR signaling could be disrupted involves inhibiting one or more AR co-activators. Bromodomain 4 (BRD4) and mixed-lineage leukemia (MLL) likely function to regulate AR activity and have both been shown to physically interact with the AR N-terminal domain [10, 18]. Importantly, inhibiting BRD4 or MLL results in an anti-tumor effect in AR signaling-competent models of CRPC. It should be noted, however, that epigenetic regulators of AR transcription are unlikely to be specific to the AR, and their inhibition may result in altering the transcriptional program across an array of bystander genes. Prostate cancer-specific studies testing MLL and bromodomain inhibitors have not begun yet, but seem likely to occur in the future based on this preclinical data.

Multiple drugs have also been shown to suppress AR-V transcripts and/or protein express. The novel AR-directed therapeutic galeterone has reportedly three different mechanisms by which can prevent AR signaling: (i) inhibiting extragonadal androgen synthesis through CYP17 inhibition (an enzyme critical to adrenal and possibly intratumoral androgen production), (ii) preventing AR nuclear translocation, and (iii) by promoting AR-FL, mutant AR, and AR-V proteosomal degradation [75, 76, 86]. Based on encouraging results from the phase I/II trial, a phase III study testing galeterone vs. enzalutamide in men with AR-V7+ CRPC is planned [87].

Similar to galeterone, the anti-helminthic drug niclosamide has been shown to potentially suppress AR-V expression [77]. Interestingly, it has no effect AR-V7 mRNA transcript levels. Instead, it has been reported to enhance AR-V7 degradation, resulting in an anti-neoplastic effect in otherwise enzalutamide-resistant prostate cancer cell lines. While this effect is modest when niclosamide is used alone, it has a synergistic effect when combined with enzalutamide, likely because niclosamide does not have effect on AR-FL. Niclosamide’s pharmacokinetics may be a barrier to developing it as a prostate cancer drug, however, as it has been found to have poor bioavailability (albeit only in one small study) [88]. As a strategy to overcome this issue of bioavailability, a phase I trial testing enzalutamide plus high-dose niclosamide in AR-V7+ men with CRPC is being pursued (clinicaltrials.gov: NCT02532114). Of note, because of niclosamide’s specificity for AR-V7, this trial may shed some light on the question of whether AR-V7 is a driver of or merely a marker for resistance. That is, if clinical activity is observed with combination treatment and AR-V7 expression is suppressed, that would speak to AR-V7 functioning as a driver of resistance. On the other hand, if there is clinical activity and AR-V7 expression is unaffected, it would seem more plausible that AR-V7 is a biomarker for a larger resistance program. If AR-V7 expression is suppressed and no clinical activity is observed, this may offer some evidence to refute AR-V7 function as a driver of resistance. Clearly, a small phase I study will not put this issue to rest, but it could provide valuable insights regarding the biology of AR-V7 nonetheless.

Preclinical models have shown that castrate androgen levels are able to rapidly induce the expression of AR-V7 and conversely that supplementation with supraphysiologic androgen concentrations can acutely suppress their expression [25, 78]. Based on additional evidence that supraphysiologic androgens are able to exert a paradoxical anti-tumor effect in models of CRPC, a pilot study testing pharmacologic doses of testosterone was launched [78]. This study documented radiographic and PSA responses in ~50 % of enrolled men. Interestingly, the authors observed a possible resensitization effect whereby following treatment with testosterone, re-challenge with an AR antagonist (i.e., enzalutamide or bicalutamide) resulted in a PSA response. Similarly, men that had previously progressed on the next-generation AR-directed therapies abiraterone and enzalutamide demonstrated high response rates upon treatment with the alternate agents—contrary to reports of clinical cross-resistance when these agents are used sequentially [6, 89–96]. This effect needs to be confirmed in larger prospective studies, but it remains possible that suppression of AR-FL and/or AR-Vs may explain why some patients appear to be *more* sensitive to drugs that inhibit AR signaling following testosterone therapy. A clinical trial specifically testing the ability of pharmacologic dose testosterone to act as re-sensitizing agent is currently underway (clinicaltrials.gov: NCT02090114).

#### Alternate nuclear hormone receptor mediated AR activation

The GR is another nuclear hormone transcription factor and has been shown to potentially drive castration-resistant cell growth through activating a similar transcriptional program to the AR [57–59]. A recently reported neoadjuvant trial testing abiraterone plus LHRH analogue vs*.* abiraterone, enzalutamide, and LHRH analogue for 6 months pre-prostatectomy in men with high-risk localized prostate cancer supports a role for both AR-V and GR signaling in conferring resistance to these potent combinatorial therapies [60]. The authors found that the loss of AR ligand binding domain, as measured by immunohistochemistry for the AR-C terminus,and increased GR expression were significantly correlated with the higher tumor epithelial volume.

Somewhat disappointingly, a small study testing the GR antagonist mifepristone did not demonstrate evidence of clinical efficacy in CRPC patients; however, the authors noted an increase in testosterone and DHT levels after 29 days of therapy—presumably due to feedback mechanism leading to increased ACTH production and in turn higher adrenal androgen biosynthesis [97]. Another strategy being explored is whether combining mifepristone with enzalutamide, to block any residual androgens from binding AR, is safe and will lead to improved outcomes (clinicaltrials.gov: NCT02012296). Whether this strategy will be effective remains to be seen; however, it should be noted that completely inhibiting GR signaling is not compatible with life, and therapeutic strategies aimed at completely inhibiting its function are not likely viable [57].

### Alternative pathway AR activation

A number of additional oncogenic signaling pathways interact with and promote persistent AR transcriptional activity [98]. Pathways commonly affected include the PI3K, WNT, JAK/STAT, and growth factor pathways. In addition, phosphoproteomic analyses have implicated a number of tyrosine kinase signaling pathways as potential prostate cancer drivers (e.g., SRC, EGFR, RET, ALK, and MAPK1/3) [15]. Many of these pathways demonstrate significant cross talk not only with the AR but also between each other—making it exceedingly difficult to develop an effective therapeutic strategy that focuses on impairing only one of the nodes within these complex signaling networks. Below is a discussion of some of the more commonly altered pathways in prostate cancer (Fig. 3).

#### PI3K pathway

Somatic alterations in the PI3K/Akt/mTOR signaling pathway occur in nearly 50 % of CRPC cases, and there is evidence that significant cross talk exists between this and the AR signaling pathway as well as other oncogenic pathways (e.g., RAS/RAF/MEK) [31, 99–102]. As evidence of the close relationship between the PI3K and AR signaling pathways, *PTEN*-deficient models of prostate cancer have revealed a reciprocal feedback relationship between these two pathways whereby suppression of AR signaling promotes PI3K pathway activation, and conversely, suppression of PI3K signaling results in AR activation [102]. Importantly, this model demonstrated that dual inhibition of both pathways resulted in a profound anti-tumor effect.

Genetic aberrations commonly occurring along the PI3K pathway include biallelic loss of *PTEN*; mutations, amplification, and activating fusions in *PIK3CA*; activating mutations in *PIK3CB*; and activating mutations in *AKT1* [31]. *PTEN* is the most commonly affected gene in this pathway and is altered in approximately 40 % of CRPC cases [103, 104]. It acts as a negative regulator of PI3K/Akt/mTOR signaling and when inactive or lost leads to pathway over activation. The PI3K signaling pathway has diverse array of functions and has been implicated in promoting prostate cancer growth, survival, proliferation, migration, stem cell-like properties and angiogenesis [98, 105–108]. Speaking to this pathways importance, *PTEN* loss has been correlated with more advanced stage and higher Gleason scores [109, 110].

There have been a number of efforts to target the PI3K/Akt/mTOR pathway, and Pan-PI3K, Akt and mTOR inhibitors are all currently in clinical testing [101, 111–117]. To date, most of the prostate cancer-specific experience targeting this pathway comes from studies testing allosteric mTOR inhibitors, with these studies all generally failing to demonstrate sufficient activity to warrant further study [111–115]. There are a number of explanations that may explain this lack of activity. For one, allosteric mTOR inhibitors only inhibit mTORC1 activity, and evidence supports mTORC2 as being an important activator of Akt in prostate cancer cells [118]. Increased reciprocal signaling through other oncogenic pathways (e.g., AR and RAS/RAF/MEK) could also render these agents ineffective. Finally, allosteric mTORC1 inhibitors do not inhibit key downstream effectors such as eIF4E, a rate-limiting initiation factor that has been implicated in mTOR-mediated translation of a number of oncogenic genes [106]. Ongoing efforts to target this pathway more effectively include developing ATP dual mTORC1/mTORC2 inhibitors, Akt inhibitors, and pan-PI3K inhibitors [101, 105, 119].

#### Epidermal growth factor pathways

Signaling through the epidermal growth factor (EGF) and its family of receptors (i.e., EGFR, HER2/Neu, erbB3, and erbB4) has also been implicated in the growth and progression of prostate cancer [120]. For instance, HER2 has been shown to be inducible in castrate conditions and to result in elevated AR target genes such as PSA [121–123]. These pathways’ effects are likely in large part mediated through MAPK activation, which can lead to phosphorylation of the AR N-terminal and subsequent ligand-independent transcription of AR target genes [24].

Based on the aforementioned observations, a handful of trials exploring the utility of targeting HER2 and/or EGFR signaling have been completed [124]. A phase II trial testing afatinib, a tyrosine kinase inhibitor (TKI) targeting EGFR and HER2, alone and in combination with nintedanib, a multi-targeted anti-angiogenic TKI, in men with CRPC was recently reported [125]. In that study, afatinib did not demonstrate any clear anti-tumor activity, with all 20 patients on the afatinib monotherapy arm progressing after 12 weeks of therapy. Lapatinib, another dual EGFR/HER-2 TKI, has also been tested in prostate cancer population [126–128]. In a single-arm phase II study targeting men with biochemically recurrent disease (i.e., non-metastatic patients with an elevated PSA), the primary endpoint of PSA_50_ response (i.e., ≥50 % decline in PSA from baseline) was not observed in any patients; however, it did appear to favorably augment PSA kinetics, resulting in a decreased PSA slope [126]. Another study in ADT-naïve patients (metastatic [5] and non-metastatic [*N* = 18]) also documented no PSA_50_ responses (the primary endpoint). Finally, in a phase II trial enrolling men with CRPC (both metastatic [*N* = 14] and non-metastatic [*N* = 7]), lapatinib was also shown to have minimal single agent activity, with only 1/21 patients achieving a PSA_50_ response (the primary endpoint) [127]. Similarly disappointing results were observed in studies testing the monoclonal anti-HER2 antibodies trastuzumab and pertuzumab in patients with CRPC [129, 130]. In total, these trials enrolled 86 patients, with none of them demonstrated a PSA_50_ response.

#### Insulin-like growth factor pathway

Similar to EGFs, the insulin-like growth factors (IGFs) have far-reaching biologic effects, promoting the growth, development, and survival of cells [131]. IGF signaling as it pertains to prostate cancer growth and progression is likely mediated through type I IGF receptor (IGF-IR) [132]. IGF-IR signaling demonstrates significant cross talk with the AR, modulating the AR transcriptional profile, AR nuclear translocation, and AR phosphorylation profile [132–134]. IGF-I levels have also been linked to a greater risk of prostate cancer progression [135–138].

Based on these observations, a randomized phase II study in men with hormone-sensitive metastatic prostate cancer comparing androgen deprivation therapy with or without cixutumumab, a monoclonal antibody targeting IGF-IR, was launched [139]. Ultimately, there was no significant difference in the rate of undetectable PSA at 28 weeks (the primary endpoint) with the addition of cixutumumab. Cixutumumab has also been studied in combination with mitoxantrone in men with CRPC and was also found to have insufficient activity to warrant further development [140]. Studies evaluating cixutumumab as part of a targeted combination strategy have yet to be reported (clinicaltrials.gov: NCT01026623, NCT00683475).

#### JAK/STAT pathway

Cytokines, particularly IL-6, have also been implicated in the maintenance of AR signaling—likely through enhanced JAK/STAT signaling. IL-6 has also been shown to associate with advanced stage prostate cancer and survival [141–144]. STAT3, one of the downstream effectors of IL-6, can lead to AR-STAT3 complex formation and subsequent AR activation [145]. Of note, EGFR signaling is also able to promote AR-STAT3 complex formation, and STAT3 has been shown to increase EGFR-mediated AR transcriptional activation—likely indicating that a complex signaling network exists between AR signaling and STAT3, IL-6, and EGFR [146].

Agents targeting this pathway include siltuximab, a monoclonal antibody targeting IL-6. In a phase I pre-surgical neoadjuvant study testing siltuximab as a monotherapy, it was shown to be well tolerated, associates with increased Ki-67 expression (a marker of apoptosis), and results in downregulation of genes downstream of IL-6 [147]. This agent was also tested in a phase I study combining it with docetaxel at the standard dose of 75 mg/m^2^ IV every 21 days [148–150]. The combination was well tolerated, and no PK interaction was noted. Not surprisingly, since it was given in combination with docetaxel, a PSA response rate of 62 % was observed. In contrast, a phase II study comparing siltuximab plus mitoxantrone vs. mitoxantrone alone was prematurely terminated due to concern for lack of efficacy at interim analysis [151].

#### WNT pathway

Wnt signaling plays an important role in early embryonic development as well as in promoting the growth and progression of several malignancies [131, 152]. Canonical Wnt signaling leads to the accumulation of β-catenin within the nucleus and leads to target gene transcription. Cross talk between the canonical Wnt and AR signaling pathways occurs through β-catenin, which can augment AR transcriptional activity [153–156]. Non-canonical Wnt signaling may also play a role in promoting prostate cancer cell survival [157]. A recent CTC transcriptome analysis has demonstrated that non-canonical Wnt signaling associates with enzalutamide drug resistance, and preclinical models suggest that WNT5A, a ligand of the non-canonical Wnt pathway, may abrogate the anti-tumor effects of AR inhibition.

Small molecule inhibitors of Wnt/β-catenin have been developed and shown to inhibit proliferation in prostate cancer cells [158]. Clinical trials evaluating specific Wnt signaling inhibitors are currently underway (clinicaltrials.gov: NCT01608867), but have yet to be reported. In addition, there is evidence that the COX-2 inhibitor celecoxib is able to inhibit Wnt signaling, and a clinical trial testing celecoxib vs*.* placebo has been reported [158, 159]. This trial was terminated early due to concerns over the cardiovascular morbidity associated with celecoxib. The celecoxib arm demonstrated only modest improvements in PSA dynamics and ultimately further development of COX-2 inhibitors as repurposed prostate cancer drugs seems unlikely.

## Conclusions

Even in its late stages, AR signaling remains an important driver of prostate cancer progression, with most of the adaptive changes that occur within a prostate cancer cell stemming from a persistent addiction to the AR. As our understanding of the alterations that occur (e.g., *AR* mutations, AR alternative splicing events, AR overexpression/copy number gains, AR substitutions, ligand persistence, and feedback pathways) in response to the evolutionary pressure exerted through chronic AR signaling inhibition, we will hopefully begin to see the emergence of therapeutic regimens that are able to impair key resistance mechanisms. Ideally, this will allow us to offer our patients a personalized regimen based on their cancer’s molecular profile and the specific resistance mechanisms operative within them.

The incredible amount of molecular redundancy by which a malignant prostate cell is able to keep AR signaling engaged remains a critical barrier to the wide deployment of targeted, precision oncology. Clinical trials targeting these accessory pathways have, to date, been mostly unsuccessful. A key issue not addressed in past trials is the issue of cross talk between pathways other than the AR. It is likely that combination therapeutic trials are necessary if these other pathways are to be effectively targeted in the clinic.

Our understanding for how prostate cancers are able to progress in spite of drugs that should effectively prevent canonical AR signaling (i.e., abiraterone and enzalutamide) is growing exponentially. Armed with this improved understanding, the next challenge will be to take this knowledge and translate it into the next generation of targeted therapies—allowing us to extend the health and well-being of men suffering with the most advanced forms of CRPC.
